# AhNRAMP1 Enhances Manganese and Zinc Uptake in Plants

**DOI:** 10.3389/fpls.2019.00415

**Published:** 2019-05-07

**Authors:** Nanqi Wang, Wei Qiu, Jing Dai, Xiaotong Guo, Qiaofang Lu, Tianqi Wang, Shiqin Li, Tongtong Liu, Yuanmei Zuo

**Affiliations:** ^1^College of Resources and Environmental Sciences, National Academy of Agriculture Green Development, Key Laboratory of Plant-Soil Interactions, Ministry of Education, China Agricultural University, Beijing, China; ^2^College of Agriculture, Ludong University, Yantai, China

**Keywords:** *AhNRAMP1*, manganese, zinc, iron, cadmium, transporter, peanut, biofortification

## Abstract

Manganese (Mn) and zinc (Zn) play essential roles in plants. Members of the natural resistance-associated macrophage protein (NRAMP) family transport divalent metal ions. In this research, the function of peanut (*Arachis hypogaea* L.) AhNRAMP1 in transporting Mn and Zn, as well as its potential for iron(Fe) and Zn biofortification was examined. *AhNRAMP1* transcription was strongly induced by Mn- or Zn-deficiency in roots and stems of peanut. Yeast complementation assays suggested that *AhNRAMP1* encoded a functional Mn and Zn transporter. Exogenous expression of *AhNRAMP1* in tobacco and rice enhanced Mn or Zn concentrations, improving tolerance to Mn or Zn deficiency. With higher Mn concentration, transgenic plants exhibited inhibited growth under Mn excess condition; similar results were obtained under excessive Zn treatment. *AhNRAMP1* expression increased biomass in transgenic tobacco and rice, as well as yield in transgenic rice grown on calcareous soil. Compared with non-transformed (NT) plants, Fe and Zn concentrations were elevated whereas concentrations of Mn, copper (Cu), and cadmium (Cd) were not enhanced. These results revealed that *AhNRAMP1* contributes to Mn and Zn transport in plants and may be a candidate gene for Fe and Zn biofortification.

## Introduction

Manganese and Zn are essential micronutrients for plant growth. Mn is not only required for photosynthesis but also acts as a cofactor of numerous enzymes (Ishimaru et al., 2012a). Mn deficiency in plants causes interveinal chlorosis and impairs many physiological processes, leading to reduced biomass (Schmidt et al., 2016). Zn is a structural component of many transcription factors and the only metal ion present in all six enzyme classes (Gupta et al., 2016). Zn deficiency leads to leaf chlorosis, photosynthesis inhibition, biomass reduction, and generation of reactive oxygen species (Sinclair and Krämer, 2012). Nevertheless, excessive Mn or Zn is harmful to plants. Excessive Mn results in oxidative stress and disturbs enzyme activity (Millaleo et al., 2010). Necrosis of old leaves and reduced biomass are also triggered by excessive Zn (Tsonev and Lidon, 2012).

Members of NRAMP family are critical metal transporters in plants. Of particular interest is the functions of NRAMP proteins in transporting divalent trace metals like Fe, Mn, and Zn because of their irreplaceable roles in many metabolic processes (Nevo and Nelson, 2006). Owing to the toxicity of Cd to humans and plants, many researchers explored the function of NRAMP proteins in transporting Cd apart from essential metals (Cailliatte et al., 2009). The five out of six *NRAMP* genes identified in the model plant *Arabidopsis thaliana* have been functionally characterized. AtNRAMP1, AtNRAMP3, and AtNRAMP4 participate in the transportation of Fe and Mn (Curie et al., 2000; Lanquar et al., 2005, 2010; Cailliatte et al., 2010). *AtNRAMP2* encodes an Mn transporter (Gao et al., 2018). AtNRAMP4 is an identified Zn transporter (Lanquar et al., 2004). AtNRAMP6 functions as an intracellular metal transporter of Cd (Cailliatte et al., 2009). In rice (*Oryza sativa*), OsNRAMP1 transports Fe and Cd, but not Mn (Takahashi et al., 2011). Whereas both OsNRAMP5 and OsNRAMP6 are Fe and Mn transporter, but OsNRAMP5 can also transport Cd (Ishimaru et al., 2012b; Sasaki et al., 2012; Peris-Peris et al., 2017). OsNRAMP3 participates in remobilization of Mn, but not that of Fe, Cd, and Zn. OsNRAMP4 do not have any role in the transportation of Fe, Mn or Cd. The functional specificity of certain NRAMP proteins is distinct and needs to be elucidated side by side.

In our preliminary research, the peanut *AhNRAMP1* was isolated by suppression subtractive hybridization. Evidence showed that AhNRAMP1 functioned as a Fe transporter localized at the plasma membrane (Xiong et al., 2012). Induced *AhNRAMP1* expression contributed to improving Fe nutrition in peanut/maize intercropping system, especially in the vegetative growth stage (Xiong et al., 2012; Dai et al., 2018). Zn concentration of peanut shoots was also enhanced by the intercropping system (Inal et al., 2007), but whether *AhNRAMP1* was involved in Zn concentration enhancement remained unknown. Besides, *AhNRAMP1* also encodes a functional Cd transporter (Chen et al., 2017). These findings propelled us to verify the functions of AhNRAMP1 in transporting Mn and Zn.

Furthermore, Fe or Zn deficiency negatively influences not only yield and quality of crops but also human health (Gupta et al., 2016; Singh et al., 2017). Biofortification is an approach to improve the mineral and vitamin values of crops utilizing plant breeding, transgenic, and agronomic practices (Bouis and Saltzman, 2017). It is essential for human health to elevate Fe and Zn concentrations in the edible tissues of crops. Overexpression of *Iron-Regulated Transport 1* (*OsIRT1*) in rice increased Fe and Zn concentrations in mature seeds and enhanced tolerance to Fe deficiency (Lee and An, 2009). Expression of *HvMTP1* (*Metal Tolerance Protein 1*), a transition metal transporter, improved Zn accumulation in barley endosperm (Menguer et al., 2018). Expression of heterologous transporter genes also improved crop Fe and Zn nutrition. Exogenous overexpression of the *MxIRT1* gene enhanced Fe and Zn concentrations in rice seeds (Tan et al., 2015). Briefly, transporter genes are indispensable to transgenic technology for their roles in Fe and Zn biofortification. Given the function of *AhNRAMP1* in transporting Fe, it is vital to investigate its Fe or Zn biofortification potential under soil condition.

In the present study, we found the AhNRAMP1 functioning as Mn and Zn transporter. *AhNRAMP1* transgenic tobacco and rice were more tolerant to Mn or Zn deficiency as compared to NT plants. Additionally, exogenous expression of *AhNRAMP1* increased the yield of rice grown on calcareous soil. Fe and Zn concentrations were higher in transgenic plants while concentrations of Mn, Cu, and Cd were not enhanced by *AhNRAMP1* expression. Consequently, we propose AhNRAMP1 as an Mn and Zn transporter and an important candidate transporter for Fe and Zn biofortification.

## Materials and Methods

### Plant Materials and Growth Conditions

Peanut (*A. hypogaea* L. cv. Luhua 14) seedlings were grown in continuously aerated nutrient solution. The nutrient solution composition was: 2.00 mM Ca(NO_3_)_2_, 0.70 mM K_2_SO_4_, 0.10 mM KCl, 0.10 mM KH_2_PO_4_, 0.50 mM MgSO_4_, 10.00 μM H_3_BO_3_, 0.50 μM MnSO_4_, 0.50 μM ZnSO_4_, 0.20 μM CuSO_4_, 0.01 μM (NH_4_)_6_Mo_7_O_24_, and 100.00 μM Fe(III)-EDTA at pH 5.8–6.0. The plants were grown in a greenhouse under a 14 h light [25°C, 300 μmol/(m^2^⋅s)] and 10 h dark (20°C) cycles. After germination in quartz sand for 14 days followed by growth in complete nutrient solution for 7 days, some peanut plants were transferred to a nutrient solution without additional Mn or Zn for 10 days as Mn or Zn depleted treatments while the others (triplicate replicates) still grown in complete nutrient solution were as a control.

### Yeast Functional Complementation

The full-length coding sequence of *AhNRAMP1* with restriction sites was amplified with the primers *Xba*I-*AhNRAMP*1-F (5′-TCTAGAATGGCAAGCGTTCTTAGACA-3′) and *Sac*I-*AhNRAMP1*-R (5′-GAGCTCTTATTCCGGTAGTGGGATAT-3′). After digestion, the *AhNRAMP1* cDNA was introduced into the yeast expression vector pDR195. The resulting pDR195-*AhNRAMP1* constructs were then transformed into the *Saccharomyces cerevisiae* strains using the LiAc/SS-DNA/PEG method (Xiong et al., 2012): The *S. cerevisiae* strains used were the Mn uptake-defective mutant *Δsmf1* (*MATα his3 ade2 leu2 trp1 ura3smf1*::URA*3ura3*::*TRP1*) (Thomine et al., 2000) and the Zn uptake-defective mutant *Δzhy6* (*MATα ade6 can1 his3 leu2 trp1 ura3 zap1Δ*::*TRP1*) (Zhao and Eide, 1997). Empty vector pDR195 was transformed into *Δsmf1* and *Δzhy6* as negative controls and the *S. cerevisiae* WT strain DEY1457 (*MAT ade6 can1 his3 leu2 trp1 ura3*; Zhao and Eide, 1997) as a positive control. The transformed cells were selected and cultured on solid synthetic dropout medium without uracil. The transformed cells were diluted to an OD at 600 nm of 0.1 to 0.00001 and spotted onto the plates containing Mn- and Zn-limiting medium. The Mn-limiting medium contained 20 mM ethylene glycol-bis-β-aminoethylether-N, N, N′, N′-tetraacetic acid (EGTA; Sigma-Aldrich, St. Louis, MO, United States). The Zn-limiting medium contained 0.1 mM EDTA (Sigma-Aldrich, St. Louis, MO, United States). The spotted yeast cells were incubated at 30°C for 2∼3 days.

### Generation of Transgenic Plants

The coding region of the *AhNRAMP1* gene was amplified using the primers 5′-TCTAGAATGGCAAGCGTTCTTAGACAG-3′ and 5′-GAGCTCTTATTCCGGTAGTGGGATATC-3′. *Xba*I and *Sac*I restriction sites were used to replace *AhNRAMP1* cDNA with the *β-glucuronidase* (*GUS*) gene of E-90Ω plasmid (Kobayashi et al., 2004). The resultant plasmid has the backbone of the pIG121Hm binary vector (Hiei et al., 1994) and drives *AhNRAMP1* cDNA under the control of the –273/–131 region of the barley *IDS2* gene containing iron-deficiency-responsive element1 (IDE1) and IDE2, flanked by the -90/+8 region of the cauliflower mosaic virus (CaMV) 35S promoter and the *Tobacco mosaic virus* 5′ leader (Ω) sequence. The constructed plasmid was introduced into *Agrobacterium tumefaciens* strain C58 by electroporation and then transformed into tobacco (*Nicotiana tabacum* L. cv. Petit-Havana SR1) using the standard leaf-disk method. E-90Ω rice lines transformation was conducted via an *Agrobacterium*-mediated method (Kobayashi et al., 2001) by using the rice cultivar *O. sativa* L. Tsukinohikari. We also generated *AhNRAMP1* transgenic rice using a plasmid driving the *GUS* gene under control of the 800 bp upstream region of the *OsIRT1* gene (Ishimaru et al., 2006). The *GUS* gene of the *OsIRT1* promoter plasmid was replaced by the *AhNRAMP1* cDNA using the *Xba*I and *Sac*I restriction sites. The above construct was introduced into *A. tumefaciens* strain C58 by electroporation. Rice transformation was conducted according to the above method described for E-90Ω rice line transformation.

### Design for Hydroponic and Pot Experiments

T_5_ transgenic E-90Ω tobacco and rice, as well as transgenic rice induced by the *OsIRT1* promoter, were generated and germinated on MS medium containing hygromycin B (50 mg/L). NT seeds were germinated on MS medium without hygromycin B. After growing 2–3 weeks for tobacco or about 4 weeks for rice in MS medium, then plants were transferred to continuously aerated nutrient solution in a greenhouse under 14 h light [25°C, 300 μmol/(m^2^⋅s)] and 10 h dark (20°C) cycles. The nutrient solution composition for tobacco was the same as described above for peanut. The rice nutrient solution consisted of 1.42 mM NH_4_NO_3_, 0.32 mM NaH_2_PO_4_⋅2H_2_O, 0.50 mM K_2_SO_4_, 1.00 mM CaCl_2_, 1.65 mM MgSO_4_⋅7H_2_O, 9.00 μM MnCl_2_⋅4H_2_O, 0.40 μM ZnSO_4_⋅7H_2_0, 0.16 μM CuSO_4_, 20.00 μM Fe(III)-EDTA, 3.00 μM H_3_BO_3_, and 0.50 μM (NH_4_)_6_Mo_7_O_24_⋅2H_2_O at pH 5.5. After growing 1 week for tobacco or 2 weeks for rice in the normal nutrient solution, the seedlings were transferred to a nutrient solution without additional Mn or Zn as nutrient deficiency treatments, or nutrient solution containing 50-times higher Mn or Zn concentration for the nutrient excess treatments. After growing 10 days for tobacco or 15 days for rice, plants were collected.

After the same progress for generation and culture on MS medium, transgenic, and NT plants were transferred to pots filled with 2 kg calcareous soil for tobacco or 6 kg for rice. The soil was collected from uncultured riverside of the Yongding river (Beijing, China). Soil pH is 8.52 with DTPA-Fe: 2.46 mg/kg, DTPA-Mn: 0.34 mg/kg, DTPA-Zn: 0.53 mg/kg, and DTPA-Cu: 0.49 mg/kg. Before being used, the soil has been amended with basal fertilizers [composition (mg/kg soil): N 100 (Ca(NO_3_)_2_⋅4H_2_O), P 150 (KH_2_PO_4_), K 100 (KCl), and Mg 50 (MgSO_4_⋅7H_2_O)]. Rice was grown under flooding condition. Tobacco plants were collected after 60 days of growth in a greenhouse with natural lighting, and rice plants were harvested after maturity.

### Analyses of Transgenic Tobacco and Rice Plants

The SPAD value of leaves was measured using a SPAD-502 chlorophyll meter (Konica-Minolta, Japan^1^). Yield composition had been recorded after rice harvested. The harvested plant samples were dried at 65°C for 3 days and weighed. Portions of ∼100 mg were digested with 4 ml of 4.4 M HNO_3_ and 2 ml of 6.5 M H_2_O_2_ for 20 min at 110°C, 20 min at 180°C, and 30 min at 220°C using a MarsXpress oven (CEM, Matthews, NC, United States^2^). Metal concentrations were measured by inductively coupled plasma atomic emission spectrometry (ICP-AES, Perkin Elmer Optima 3300DV).

### Quantitative Real-Time PCR

Total RNA was extracted using TRIzol reagent (TaKaRa, Tokyo, Japan). Genomic DNA elimination and the first cDNA synthesis from RNA was achieved using a PrimeScript^TM^ RT reagent kit with gDNA Eraser (TaKaRa, Tokyo, Japan). qPCR was performed using a KAPA SYBR fast qPCR kit (Kapa Biosystems, Inc., Woburn, MA, United States) and a qTOWER 2.2 Real-time System (Analytik Jena, Jena, German) with primers listed in Supplementary Table S1. The *AhNRAMP1* transcript quality was normalized against peanut *AhUbiquitin*. The *AhNRAMP1* transcript level was normalized against *NtActin* for transgenic tobacco and *OsActin* for rice.

### Statistical Analysis

Statistical analyses were conducted using R version 3.5.0. The statistical significance of differences between two groups was determined by Student’s *t*-Test (^∗^*p* ≤ 0.05, ^∗∗^*p* ≤ 0.01, and ^∗∗∗^*p* ≤ 0.001). Differences among multiple groups were assessed by One-way ANOVA followed by *post hoc* using Duncan’s test (*p* ≤ 0.05) when data met normal distribution and homogeneity of variance. Otherwise, non-parametric multiple comparisons by Kruskal–Wallis test (*p* ≤ 0.05) were used.

## Results

### *AhNRAMP1* Was Induced by Mn or Zn Deficiency in Both Roots and Stems of Peanut

**FIGURE 1:**
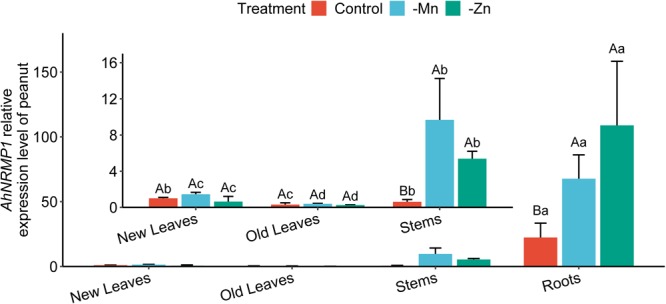
Expression patterns of *AhNRAMP1* response to Mn or Zn deficiency in peanut. Peanut plants were grown in the hydroponic system with complete nutrient solution (control) or without Mn, or Zn for 10 days. The results are presented as the mean ±SD of triplicate replicates. Different lowercase letters represent significant differences among the organs of peanut under the same treatment by Kruskal–Wallis test at *p* ≤ 0.05 while uppercase letters represent significant differences among different treatments of the same organ by Duncan’s test at *p* ≤ 0.05.

*AhNRAMP1* expression levels were investigated in different peanut organs under complete culture solution (control), as well as Mn or Zn depleted conditions. With all treatments, *AhNRAMP1* expression in stems was lower than that in roots but was higher than that in leaves (Figure 1). *AhNRAMP1* transcript level in roots was significantly increased by three-fold after treatment without Mn for 10 days compared with control (Figure 1). Additionally, *AhNRAMP1* expression was significantly upregulated (4.8-fold) in Zn-deficient roots (Figure 1). In stems, the mRNA level of *AhNRAMP1* was 15.6 times higher under Mn depleted condition and was 8.9 times higher under Zn depleted condition compared with control (Figure 1). By contrast, no changes were induced by Mn or Zn deficiency in new and old leaves (Figure 1).

### AhNRAMP1 Functionally Complemented the Growth Defect of Mn or Zn Uptake-Defective Yeast Mutants

**FIGURE 2:**
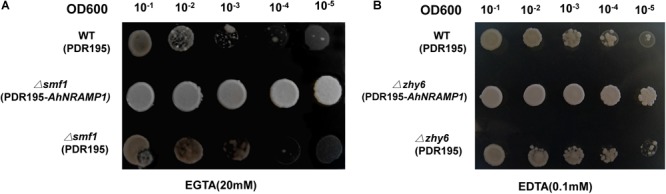
Functional complementation of the *Δsmf1* yeast mutant with *AhNRAMP1* under Mn-limiting medium **(A)** and that of the *Δzhy6* yeast mutant with *AhNRAMP1* under Zn-limiting medium **(B)**. Yeast mutants defective in the uptake of Mn (*Δsmf1*) and Zn (*Δzhy6*) transformed with empty pDR195 vector were used as negative controls, and the WT yeast DEY1457 strains transformed with empty pDR195 vector was used as positive controls. Serial dilutions of transformed yeast cells with OD_600nm_ of 0.1–0.00001 were plated on Synthetic Drop-out medium lacking Mn **(A)** or Zn **(B)** and grown for 2–3 days at 30°C.

A yeast functional complementation experiment was conducted using the *Δsmf1* (Mn uptake-defective) and *Δzhy6* (Zn uptake-defective) yeast mutants to determine the Mn and Zn transport activity of AhNRAMP1. The growth of yeast mutants expressing *AhNRAMP1* was significantly improved compared with that of yeast transformed with empty vector when grown on Mn or Zn limiting medium (Figure 2). The results inferred that AhNRAMP1 could complement *Δsmf1* and *Δzhy6* mutants and transport Mn and Zn.

### Exogenous Expression of *AhNRAMP1* Promoted Mn Uptake in Plants

**FIGURE 3:**
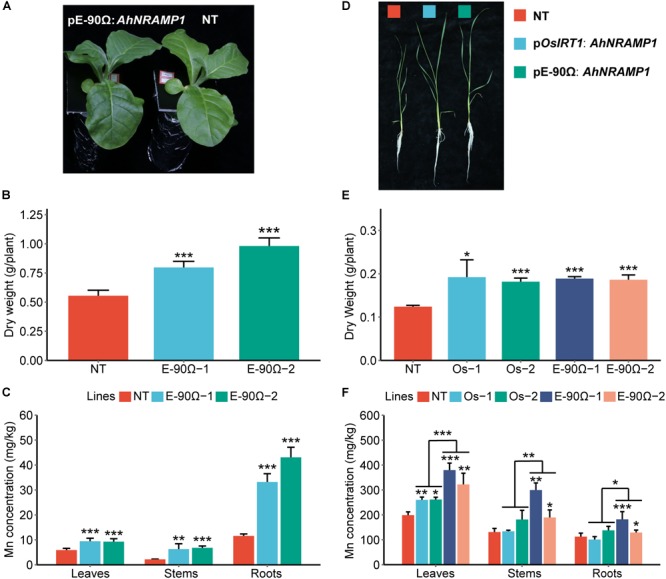
The response to Mn deficiency in *AhNRAMP1* transgenic plants. The phenotype **(A)**, biomass **(B)**, and Mn concentration **(C)** of tobacco (left, **A–C**) under Mn depleted condition for 10 days, as well as those of rice under Mn depleted condition for 15 days (right, **D–F**). NT, non-transformed plants; E-90Ω, overexpressing *AhNRAMP1* transgenic plants induced by the E-90Ω promoter; Os, *AhNRAMP1* transgenic rice induced by the *OsIRT1* promoter. The results are presented as the means ± SD of triplicate replicates. Asterisks indicate significant differences of transgenic plants with NT plants or between E-90Ω rice and *OsIRT1* promoter rice by Student’s *t*-Test (^∗^*p* ≤ 0.05, ^∗∗^*p* ≤ 0.01, and ^∗∗∗^*p* ≤ 0.001).

To investigate the physiological role of *AhNRAMP1* in plants, *AhNRAMP1* was genetically transformed into tobacco and rice, the model dicot and monocot plant, respectively (Figures 7A,B). Mn concentration was significantly higher in all organs of E-90Ω lines after Mn depleted treatment (Figures 3C,F), as a result of higher expression of *AhNRAMP1* in roots (Figures 7A,E). Biomass was also increased in E-90Ω lines (Figures 3A,B,D,E) along with the Mn content as expected (Supplementary Figure S1). Compared with NT rice under Mn depleted condition, more biomass, higher leaf Mn concentration (Figures 3D–F) and higher Mn contents (Supplementary Figure S1B) were also observed in *OsIRT1* promoter lines. The primary expression of *AhNRAMP1* in roots of *OsIRT1* promoter lines may explain the improvement (Figure 7B).

**FIGURE 4:**
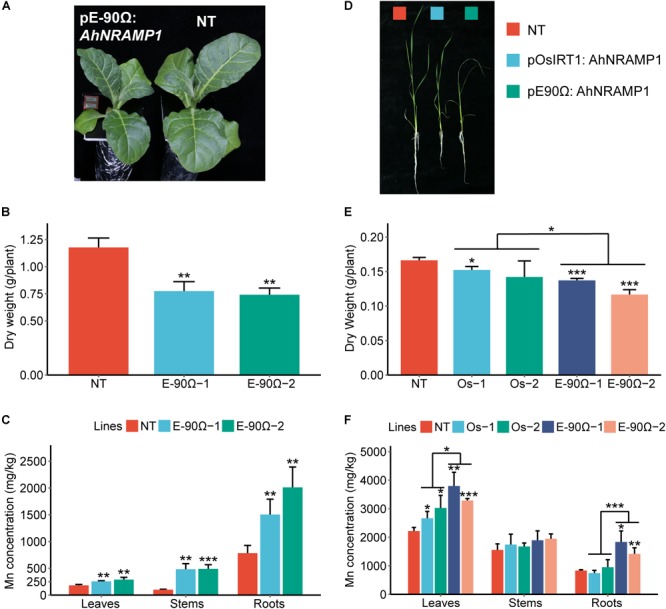
The response to excessive Mn in *AhNRAMP1* transgenic plants. The phenotype **(A)**, biomass **(B)**, and Mn concentration **(C)** of tobacco (left, **A–C**) under Mn excess condition (25 μm) for 10 days, as well as those of rice (right, **D–F**) under Mn excess condition (45 μm) for 15 days. NT, non-transformed plants; E-90Ω, overexpressing *AhNRAMP1* transgenic plants induced by the E-90Ω promoter; Os, *AhNRAMP1* transgenic rice induced by the *OsIRT1* promoter. The results are presented as the means ± SD of triplicate replicates. Asterisks indicate significant differences of transgenic plants with NT plants or between E-90Ω rice and *OsIRT1* promoter rice by Student’s *t*-Test (^∗^*p* ≤ 0.05, ^∗∗^*p* ≤ 0.01, and ^∗∗∗^*p* ≤ 0.001).

Even under Mn excess condition, the concentration of Mn was still enhanced in all organs of E-90Ω tobacco, as well as in the leaves and roots of E-90Ω rice compared with NT plants (Figures 4C,F). Biomass production was subsequently reduced in E-90Ω lines (Figures 4A,B,D,E). *OsIRT1* promoter lines also showed higher leaf Mn concentration and lowered dry weight (Figures 4D–F). Mn content was elevated in only one E-90Ω line compared with NT plants (Supplementary Figure S2). Moreover, *AhNRAMP1* expression profiles in E-90Ω lines were similar between Mn depleted and Mn excess conditions (Figures 7A–D). By contrast, *AhNRAMP1* expression level in roots of *OsIRT1* promoter lines was lower than that in leaves under Mn excess condition (Figure 7D).

Besides, *AhNRAMP1* expression in all organs of E-90Ω rice was invariably higher than that of *OsIRT1* promoter lines under Mn depleted condition (Figure 7B) and excess condition (Figure 7D). Accordingly, with higher Mn concentration, E-90Ω rice was more tolerant to Mn deficiency but more sensitive to excessive Mn compared with *OsIRT1* promoter lines (Figure 3D–F, 4D–F). Even the more severely curled leaves were observed in E-90Ω rice under Mn excess condition (Figure 4D).

### Exogenous Expression of *AhNRAMP1* Enhanced Zn Uptake in Plants

**FIGURE 5:**
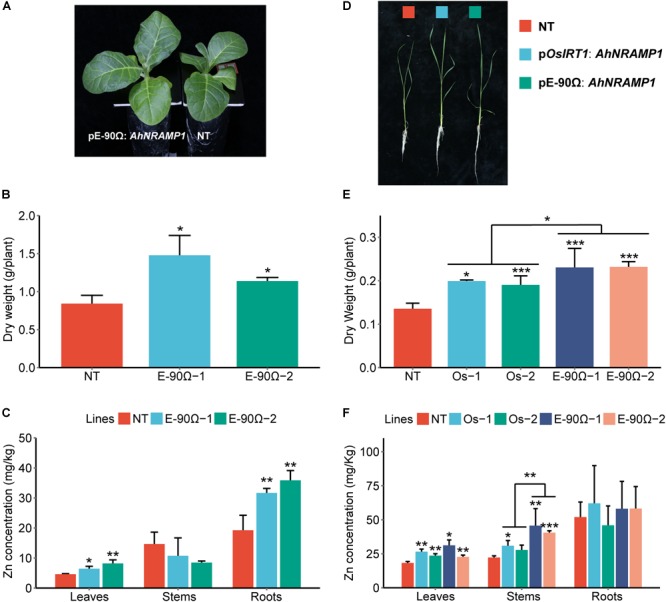
The response to Zn deficiency in *AhNRAMP1* transgenic plants. The phenotype **(A)**, biomass **(B)**, and Zn concentration **(C)** of tobacco (left, **A–C**) under Zn depleted condition for 10 days, as well as those of rice under Zn depleted condition for 15 days (right, **D–F**). NT, non-transformed plants; E-90Ω, overexpressing *AhNRAMP1* transgenic plants induced by the E-90Ω promoter; Os, *AhNRAMP1* transgenic rice induced by the *OsIRT1* promoter. The results are presented as the means ±SD of triplicate replicates. Asterisks indicate significant differences of transgenic plants with NT plants or between E-90Ω rice and *OsIRT1* promoter rice by Student’s *t*-Test (^∗^*p* ≤ 0.05, ^∗∗^*p* ≤ 0.01, and ^∗∗∗^*p* ≤ 0.001).

Higher expression of *AhNRAMP1* in roots (Figures 7E,F) increased Zn concentration of the leaves and roots in E-90Ω tobacco, as well as enhanced leaf and stem Zn concentration in E-90Ω rice under Zn depleted condition (Figures 5C,F). Leaf Zn concentration was also higher in *OsIRT1* promoter lines than NT rice (Figure 5F). As a result, more biomass production (Figures 5A,B,D,E) and higher Zn content (Supplementary Figure S3) were also detected in all transgenic plants.

**FIGURE 6:**
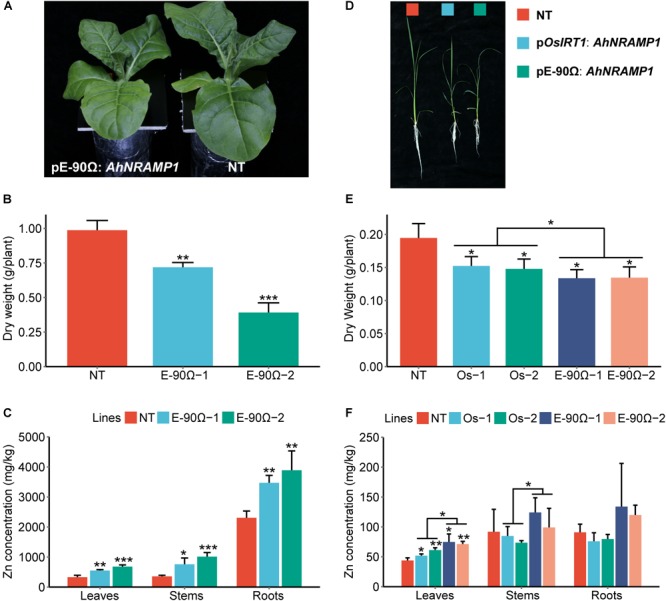
The response to excessive Zn in *AhNRAMP1* transgenic plants. The phenotype **(A)**, biomass **(B)**, and Zn concentration **(C)** of tobacco (left, **A–C**) under Zn excess condition (25 μm) for 10 days, as well as those of rice (right, **D–F**) under Zn excess condition (20 μm) for 15 days. NT, non-transformed plants; E-90Ω, overexpressing *AhNRAMP1* transgenic plants induced by the E-90Ω promoter; Os, *AhNRAMP1* transgenic rice induced by the *OsIRT1* promoter. The results are presented as the means ±SD of triplicate replicates. Asterisks indicate significant differences of transgenic plants with NT plants or between E-90Ω rice and *OsIRT1* promoter rice by Student’s *t*-Test (^∗^*p* ≤ 0.05, ^∗∗^*p* ≤ 0.01, and ^∗∗∗^*p* ≤ 0.001).

Even after excessive Zn treatment, significantly higher Zn concentration of all organs of transgenic tobacco and increased leaf Zn concentration in transgenic rice was observed (Figures 6C,F). Biomass of all transgenic lines was decreased (Figures 6A,B,D,E). There was no statistical difference in Zn content of the whole plant among different *AhNRAMP1* transgenic lines and NT plants (Supplementary Figure S4).

**FIGURE 7:**
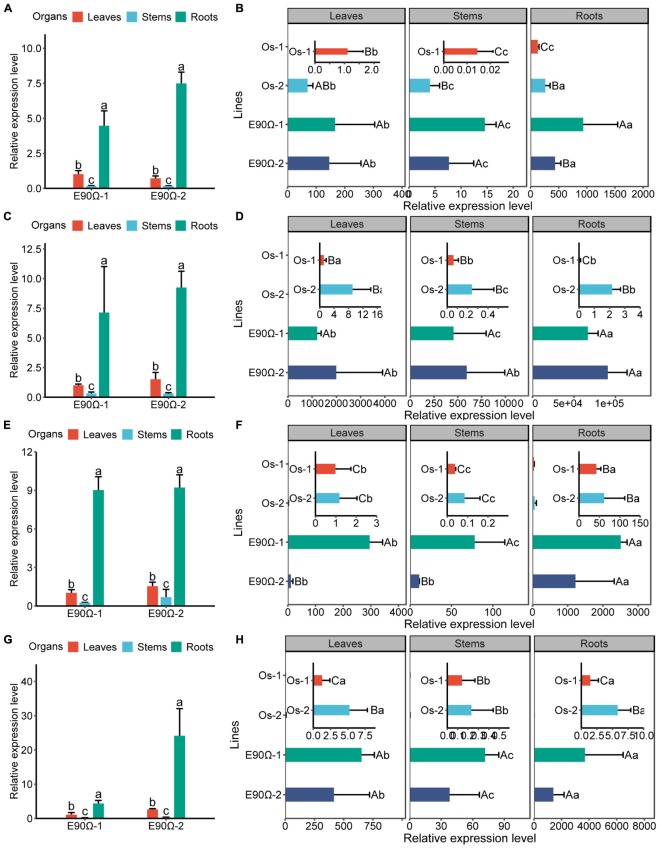
*AhNRAMP1* expression profiles of *AhNRAMP1* transgenic plants grown in the hydroponic system. *AhNRAMP1* expression level in different organs of transgenic tobacco **(A)** or rice **(B)** under Mn depleted conditions, as well as those under Mn excess conditions **(C,D)**. *AhNRAMP1* expression level in different organs of transgenic tobacco **(E)** or **(F)** under Zn depleted conditions, as well as those under Zn excess conditions **(G,H)**. NT, non-transformed plants; E-90Ω, overexpressing *AhNRAMP1* transgenic plants induced by the E-90Ω promoter; Os, *AhNRAMP1* transgenic rice induced by the *OsIRT1* promoter. The results are presented as the means ± SD of triplicate replicates. Different lowercase letters represent significant differences among organs of the same transgenic line while uppercase letters indicate significant differences among different transgenic lines of the same organ at *p* ≤ 0.05 by Duncan’s test or by Kruskal–Wallis test.

Under Zn depleted condition (Figure 7E) or excess condition (Figure 7H), *AhNRAMP1* expression level was also higher in E-90Ω rice lines than *OsIRT1* promoter lines (Figures 7F,H). Hence, E-90Ω rice lines exhibited more tolerance to Zn deficiency but more sensitivity to excessive Zn (Figure 5D–F, 6D–F). E-90Ω rice displayed more severely curled leaves under Zn excess condition (Figure 6D).

### Exogenous *AhNRAMP1* Expression Improved Fe and Zn Nutrition in Plants Grown on Calcareous Soil

**FIGURE 8:**
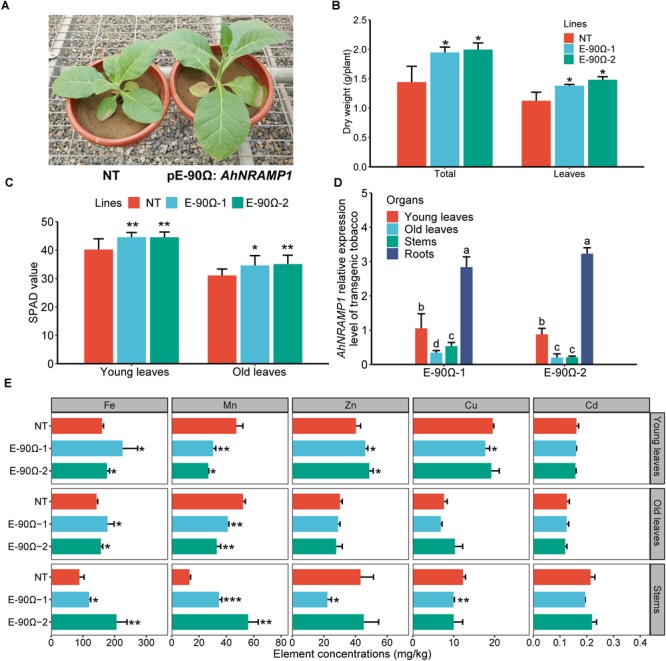
Exogenous *AhNRAMP1* improved Fe and Zn deficiencies in transgenic tobacco grown on calcareous soil. The phenotype **(A)**, biomass **(B)**, SPAD value **(C)**, *AhNRAMP1* expression profiles **(D)**, and element concentrations **(E)** of tobacco in the vegetative period. NT, non-transformed plants; E-90Ω, overexpressing *AhNRAMP1* transgenic plants induced by the E-90Ω promoter. The results are presented as the means ± SD of twelve replicates for SPAD value, and triplicate replicates for other data. Different lowercase letters represent significant differences of *AhNRAMP1* expression level among organs at *p* ≤ 0.05 by Kruskal–Wallis test. Asterisks indicate significant differences with NT plants by Student’s *t*-Test (^∗^*p* ≤ 0.05, ^∗∗^*p* ≤ 0.01, and ^∗∗∗^*p* ≤ 0.001).

For transgenic tobacco grown on calcareous soil for 60 days, biomass production and SPAD value were enhanced by higher Fe concentration of leaves and stems, as well as higher Zn concentration of young leaves (Figures 8A–C,E). The enhancement might be a consequence of the overexpression of *AhNRAMP1* in roots (Figure 8D). Stem Mn concentration was higher, but leaf Mn concentration was lower in transgenic tobacco than those in NT plants (Figure 8E). Overexpression *AhNRAMP1* did not elevate Cu and Cd concentrations in transgenic tobacco (Figure 8E). The Fe and Zn contents in the aboveground of transgenic tobacco were higher than those of NT plants (Supplementary Figure S5).

**FIGURE 9:**
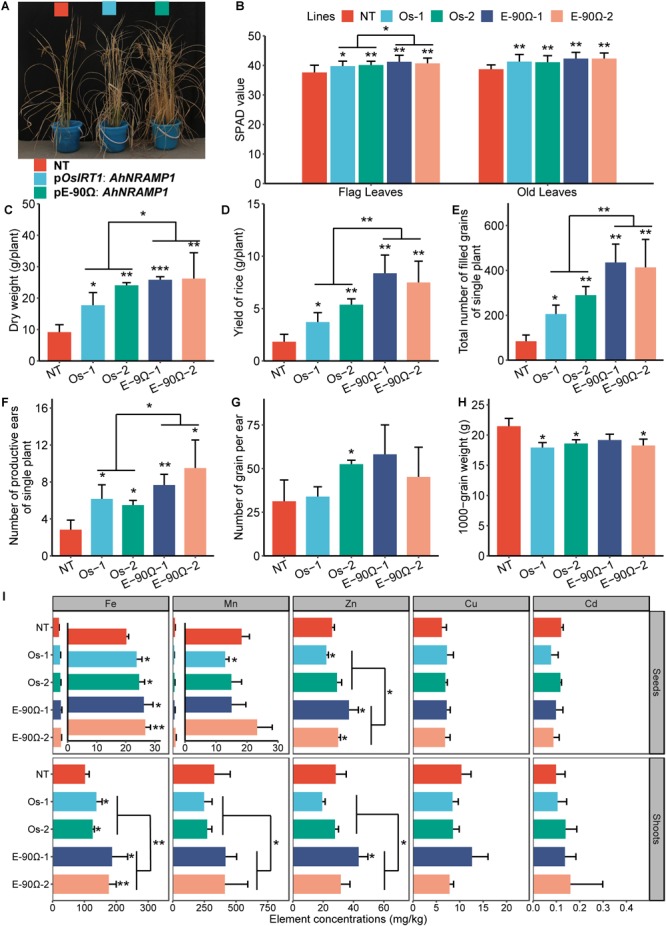
Exogenous *AhNRAMP1* improved Fe and Zn deficiencies in transgenic rice grown on calcareous soil. The phenotype **(A)**, biomass **(C)**, yield composition **(D–H)** and element concentrations **(I)** of mature rice, as well as the SPAD value of the leaves during the tillering stage **(B)**. NT, non-transformed plants; E-90Ω, overexpressing *AhNRAMP1* transgenic plants induced by the E-90Ω promoter; Os, *AhNRAMP1* transgenic rice induced by the *OsIRT1* promoter. The results are presented as the means ±SD of twelve replicates for the SPAD value, and triplicate replicates for other data. Asterisks indicate significant differences with NT plants or between E-90Ω rice and *OsIRT1* promoter rice by Student’s *t*-Test (^∗^*p* ≤ 0.05, ^∗∗^*p* ≤ 0.01, and ^∗∗∗^*p* ≤ 0.001).

SPAD value, especially in flag leaves, were higher during the tillering stage and biomass production increased after maturity in *AhNRAMP1* transgenic rice compared with NT plants (Figure 9A–C). Exceedingly more productive ears and filled grains, as well as mildly lighter 1000-grain weight, were combined to result in the higher yield of transgenic rice (Figures 9D–H). Fe concentration was higher in the seeds and shoots of all transgenic rice than NT plants (Figure 9I). Zn concentration in the seeds and shoots was higher in overexpressing E-90Ω lines (Figure 9I). Mn, Cu, and Cd concentrations were not enhanced by exogenous expression of *AhNRAMP1* (Figure 9I). Moreover, Zn concentration of seeds, Fe, Mn, and Zn concentrations of shoots, SPAD value of flag leaves, dry weight, productive eras, filled grains, and yield were higher in E-90Ω rice compared with *OsIRT1* promoter lines (Figures 9A–F,I).

## Discussion

### AhNRAMP1 Functioned as Mn and Zn Transporter in Plants

Mn and Zn are essential micronutrients for plant growth and development. Members of the NRAMP family are functional divalent metal ion transporters (Nevo and Nelson, 2006). Numerous NRAMP proteins have been identified as Mn transporters in plants (Cailliatte et al., 2010; Peris-Peris et al., 2017; Shao et al., 2017; Gao et al., 2018). Zn transport in plants is mainly conducted by members of seven transporter families excluding NRAMP (Gupta et al., 2016). The literature on how NRAMP proteins function to transport Zn in plants is very limited. Only AtNRAMP4 and TcNRAMP4 have been reported to transport Zn (Lanquar et al., 2004; Oomen et al., 2009). The function of AhNRAMP1 in Fe and Cd transport is well established (Xiong et al., 2012; Chen et al., 2017). Perhaps, AhNRAMP1 might be a functional transporter of other divalent metal ions such as Mn and Zn. *AhNRAMP1* expression was markedly upregulated in roots and stems under Mn or Zn deficient conditions (Figure 1). *AhNRAMP1* expression restored the growth of the yeast mutant *Δsmf1* defective in Mn uptake and *Δzhy6* detective in Zn uptake (Figure 2), uncovering the Mn and Zn transporting roles of AhNRAMP1.

Constitutive overexpression of *NRAMP* genes can cause a growth defect (Ishimaru et al., 2012a). Thus, an artificial promoter E-90Ω was used to drive high *AhNRAMP1* expression in roots of both tobacco and rice (Xiong et al., 2012). *OsIRT1* promoter was also used to generate transgenic rice because *OsIRT1* expression was induced in rice roots by Mn- or Zn- deficiency (Lee and An, 2009). In the hydroponic system, Mn concentration of *AhNRAMP1* transgenic plants was higher than NT plants under Mn depleted or excess conditions (Figure 3, 4). Exogenous *AhNRAMP1* expression also resulted in higher Zn concentration under Zn depleted or excess conditions (Figure 5, 6). As a result, *AhNRAMP1* transgenic plants were more resistant to Mn or Zn deficiency, but more sensitive to Mn or Zn excess conditions (Figure 3–6).

Furthermore, *AhNRAMP1* expression level in E-90Ω rice was higher than that in *OsIRT1* promoter lines under all hydroponic treatments (Figure 7). Thus, the higher expression level of *AhNRAMP1* in transgenic plants, the higher Mn or Zn concentrations were detected (Figures 3–6). As a whole, our findings indicated that AhNRAMP1 was a functional transporter of Mn and Zn in plants.

### *AhNRAMP1* May Be a Candidate Gene for Fe and Zn Biofortification

AhNRAMP1 functioned as Fe (Xiong et al., 2012), Mn, Zn (Figures 2–6), and Cd (Chen et al., 2017) transporters in plants. Accordingly, it is necessary to dissect the role of AhNRAMP1 systematically considering its role in transporting all these metals. Further, Fe and Zn deficiencies frequently disrupted agricultural production (Gupta et al., 2016; Singh et al., 2017). Therefore, we carried out pot experiments using calcareous soil, which frequently causes Fe or Zn deficiency in plants by high pH and high bicarbonate (Marschner and Röhmeld, 1994; Gupta et al., 2016). Expression of *AhNRAMP1* improved Fe and Zn nutrition (Figure 8E) in transgenic tobacco, resulting in strikingly enhanced chlorophyll contents, especially in the young leaves (Figure 8C). As poorly movable elements, Fe or Zn deficiency caused chlorosis in young leaves (Sinclair and Krämer, 2012; Xiong et al., 2012). Hence, *AhNRAMP1* transgenic tobacco exhibited more tolerance to Fe and Zn deficiency when grown on calcareous soil (Figure 8).

Biomass production was higher in *AhNRAMP1* transgenic rice (Figures 9A,C) than NT rice, which was the primary reason of higher yield (Figure 9D; Sakamoto et al., 2006). Besides, another crucial reason behind higher yield in transgenic rice was increased filled grains, which may be due to the enhancement of chlorophyll contents during the tillering stage, especially in flag leaves (Figures 9B–F). After all, flag leaves are the paramount source organs of carbohydrates production (Zhang et al., 2015). Moreover, flag leaves, as youngest leaves, readily suffer from Fe, and Zn deficiency when grown on calcareous soil (Marschner and Röhmeld, 1994; Gupta et al., 2016). From this it follows that the improvement of transgenic rice mentioned above might also be caused by elevated Fe and Zn uptake (Figure 9I and Supplementary Figure S6). Furthermore, breeding crop with an increased ability to accumulate Fe and Zn in their edible portions is beneficial to human health (White and Broadley, 2009). It is exciting that *AhNRAMP1* expression enhanced seed Fe concentration in all transgenic rice and seed Zn concentration in E-90Ω rice as compared with NT plants. These results implied that *AhNRAMP1* has the potential of Fe and Zn biofortification.

Excessive Mn or Zn is detrimental to plants, which is universal owing to environmental pollution or application of Mn- or Zn- containing fertilizers or pesticides (Millaleo et al., 2010; Tsonev and Lidon, 2012). Nonetheless, Zn is hardly excessive in plants grown on calcareous soil due to its low availability (Sadeghzadeh, 2013). Cd is one of the most toxic heavy metals to both plants and humans (Järup and Åkesson, 2009). AhNRAMP1 functioned as Mn (Figures 2–4) and Cd (Chen et al., 2017) transporters, it is essential to explore how its exogenous expression influence on Mn and Cd uptake in transgenic plants grown on calcareous soil. In our research, concentrations of Mn and Cd were enhanced neither in the leaves of transgenic tobacco nor in the seeds and shoots of transgenic rice by *AhNRAMP1* expression when grown on calcareous soil (Figures 8E, 9I). However, even under the excessive Mn or Zn supply in hydroponics, *AhNRAMP1* expression still promoted Mn or Zn concentration in transgenic plants (Figures 4, 6). The results indicated the risk of heavy metal accumulation when *AhNRAMP1* transgenic plants were grown under the provision of excessive heavy metals. Thus, a series of field experiments should be performed in future to make better use of *AhNRAMP1*, including drawing on the advantage of alleviating Fe or Zn deficiency and avoiding the disadvantage associated with excessive Mn and Cd.

## Conclusion

In conclusion, the present study provides new insights into our understanding of the *AhNRAMP1* gene. AhNRAMP1 transported Mn and Zn, and its expression was strongly induced by Mn or Zn deficiency in roots and stems of peanut. Exogenous expression of *AhNRAMP1* in tobacco and rice increased Mn or Zn concentrations. Consequently, transgenic plants exhibited improved tolerance to Mn or Zn deficiency but more sensitivity to Mn or Zn excess. Expression of *AhNRAMP1* enhanced biomass in transgenic tobacco and rice and yield in transgenic rice grown on calcareous soil. Moreover, Fe and Zn concentrations were higher and Mn, Cu, and Cd concentrations were not enhanced in *AhNRAMP1* transgenic plants compared with NT plants. *AhNRAMP1* may also be a candidate gene for Fe and Zn biofortification.

## Author Contributions

NW and YZ designed the experiments. NW and WQ performed most of the experiments. XG performed the yeast complementation tests. JD, QL, TW, SL, and TL participated in part of the plant analysis. NW wrote the manuscript. WQ, JD, and YZ revised the manuscript. All authors discussed the results and commented on the manuscript. YZ provided funding for this work as the corresponding author.

## Conflict of Interest Statement

The authors declare that the research was conducted in the absence of any commercial or financial relationships that could be construed as a potential conflict of interest.

## Footnotes

1 http://www.konicaminolta.com

2 http://www.cem.com/

## Abbreviations

Cd: Cadmium
cDNA: complementary DNA
Cu: Copper
E-90Ω: *AhNRAMP1* transgenic lines induced by the E-90Ω promoter
EDTA: Ethylenediaminetetraacetic acid
EGTA: Ethylene glycol-bis-β-aminoethylether-N
N, N′: N′-tetraacetic acid
Fe: Iron
IRT: iron-regulated transporter
Mn: Manganese
MS: Murashige and Skoog
NRAMP: natural resistance-associated macrophage protein
NT: non-transformed
Os: *AhNRAMP1* transgenic lines induced by the *OsIRT1* promoter
qPCR: quantitative real-time PCR
SD: standard deviation
WT: wild-type
Zn: Zinc
