# Supplementary material for: AhNRAMP1 Enhances Manganese and Zinc Uptake in Plants
Source: Front Plant Sci. 2019 May 7;10:415. doi: 10.3389/fpls.2019.00415 (PMC6514220; doi:10.3389/fpls.2019.00415)
Supplement: Supplementary file 1 [file Table_1.DOCX]

Supplementary Material

AhNRAMP1 enhances manganese and zinc uptake in plants

**Nanqi Wang^1,a^ and Wei Qiu^1,a^, Jing Dai^1^, Xiaotong Guo^2^, Hongchun Xiong^3^, Qiaofang Lu^1^ Tianqi Wang^1^, Shiqin Li^1^ Tongtong Liu^1^ and Yuanmei Zuo^1*^**

*** Correspondence:** Prof. Yuanmei Zuo: zuoym@cau.edu.cn

# Supplementary Figures and Tables

## Supplementary Figures


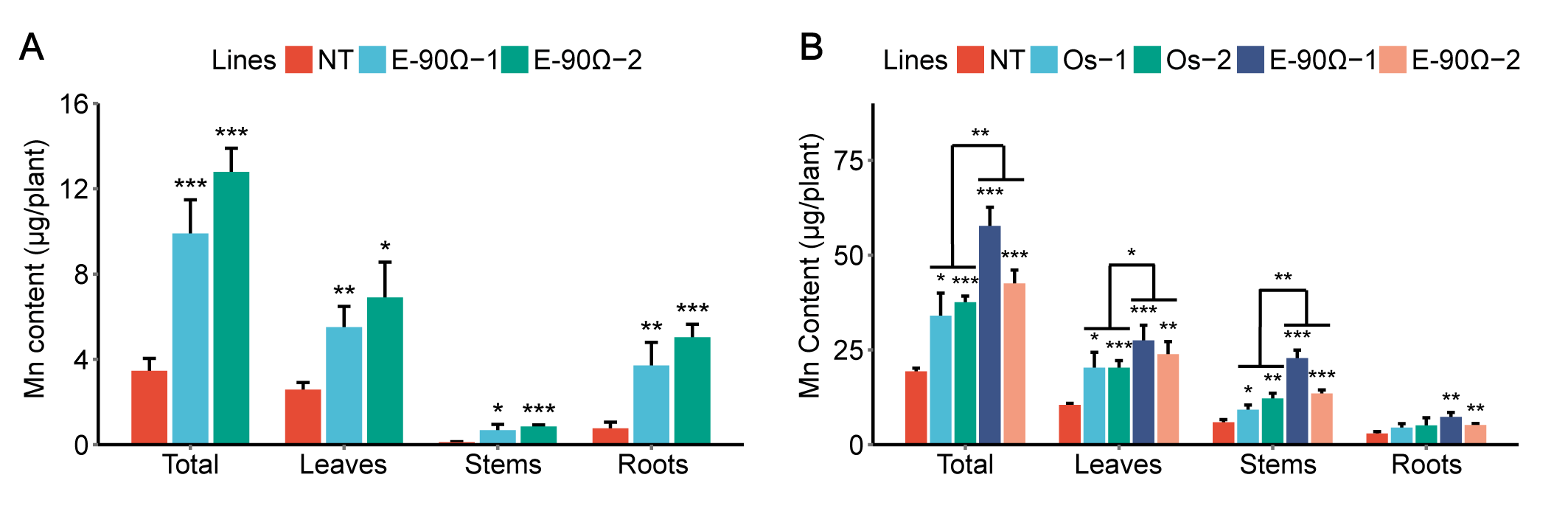


**Supplementary Figure 1.** Mn content of tobacco (A) under Mn depleted condition for 10 days, as well as that of rice under Mn depleted condition for 15 days. NT, non-transformed plants; E-90Ω, overexpressing *AhNRAMP1* transgenic plants induced by the E-90Ω promoter; Os, *AhNRAMP1* transgenic rice induced by the *OsIRT1* promoter. The results are presented as the means ± SD of triplicate replicates. Asterisks indicate statistically significant differences with NT plants or between E-90Ω lines and *OsIRT1* promoter lines according to Student’s *t*-tests: **p* ≤ 0.05, ***p* ≤ 0.01, and ****p* ≤ 0.001.


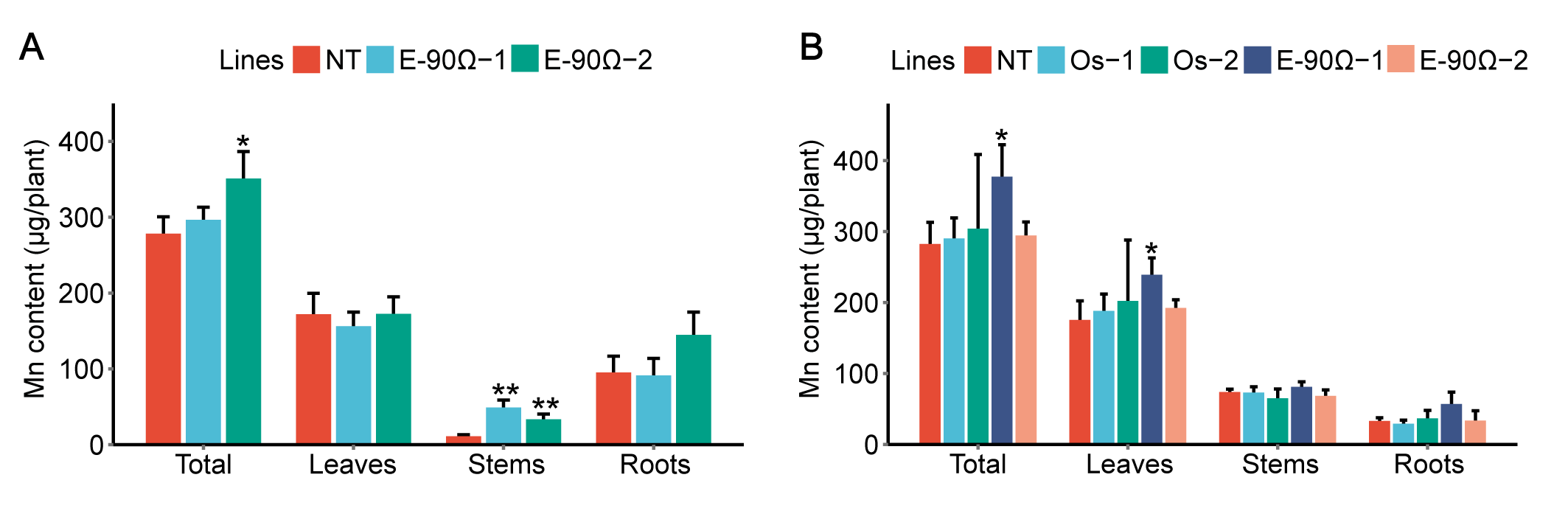


**Supplementary Figure 2.** Mn content of tobacco (A) under Mn excess condition (25 μm) for 10 days, as well as that of rice under Mn excess condition (45 μm) for 15 days. NT, non-transformed plants; E-90Ω, overexpressing *AhNRAMP1* transgenic plants induced by the E-90Ω promoter; Os, *AhNRAMP1* transgenic rice induced by the *OsIRT1* promoter. The results are presented as the means ± SD of triplicate replicates. Asterisks indicate statistically significant differences with NT plants or between E-90Ω lines and *OsIRT1* promoter lines according to Student’s *t*-tests: **p* ≤ 0.05, ***p* ≤ 0.01, and ****p* ≤ 0.001.

**Supplementary Figure 3.** Zn content of tobacco (A) under Zn depleted condition for 10 days, as well as that of rice under Zn depleted condition for 15 days. NT, non-transformed plants; E-90Ω, overexpressing *AhNRAMP1* transgenic plants induced by the E-90Ω promoter; Os, *AhNRAMP1* transgenic rice induced by the *OsIRT1* promoter. The results are presented as the means ± SD of triplicate replicates. Asterisks indicate statistically significant differences with NT plants or between E-90Ω lines and *OsIRT1* promoter lines according to Student’s *t*-tests: **p* ≤ 0.05, ***p* ≤ 0.01, and ****p* ≤ 0.001.


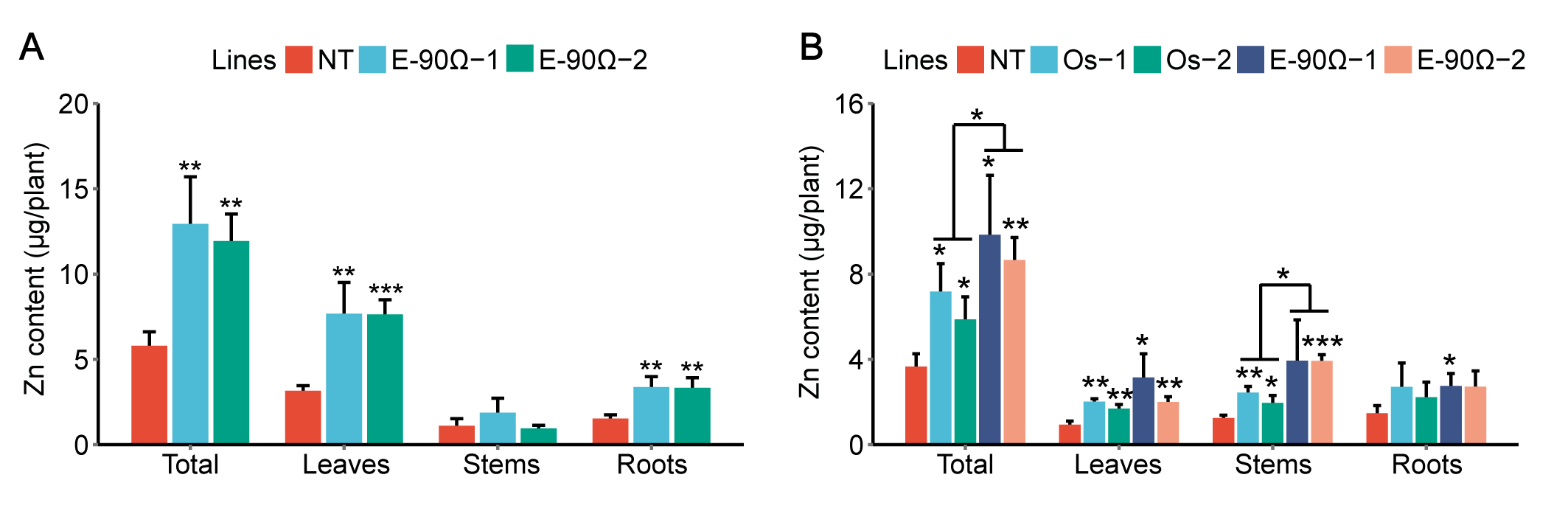

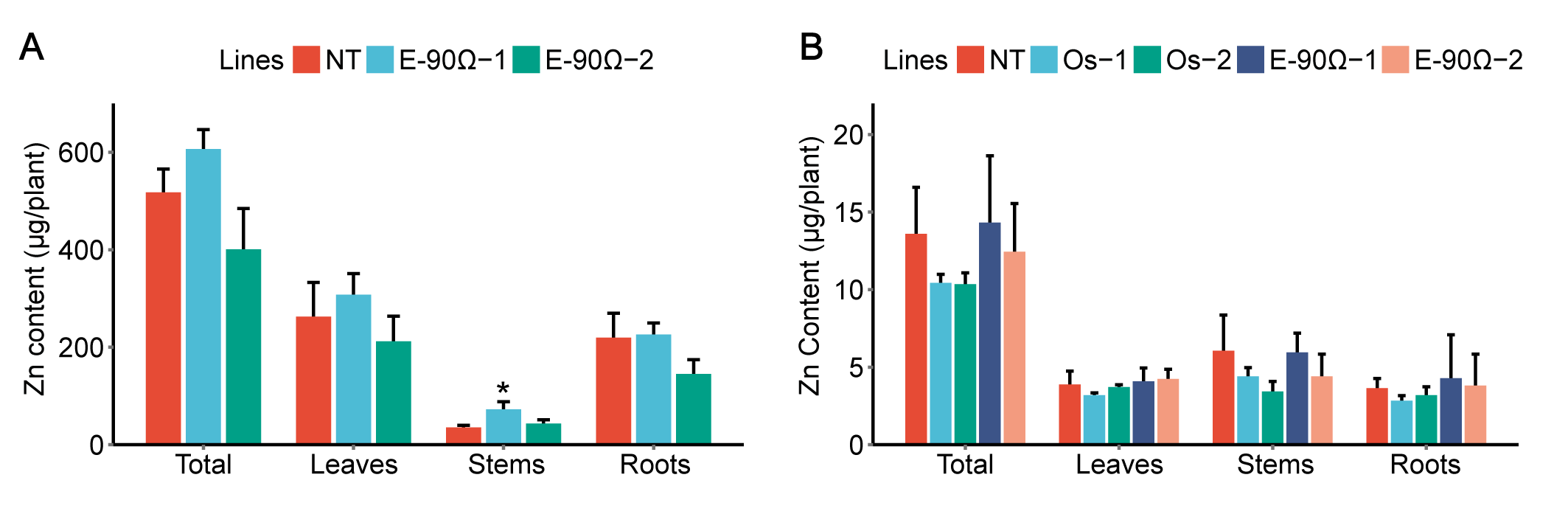


**Supplementary Figure 4.** Zn content of tobacco (A) under Zn excess condition (25 μm) for 10 days, as well as that of rice under Zn excess condition (20 μm) for 15 days. NT, non-transformed plants; E-90Ω, overexpressing *AhNRAMP1* transgenic plants induced by the E-90Ω promoter; Os, *AhNRAMP1* transgenic rice induced by the *OsIRT1* promoter. The results are presented as the means ± SD of triplicate replicates. Asterisks indicate statistically significant differences with NT plants or between E-90Ω lines and *OsIRT1* promoter lines according to Student’s *t*-tests: **p* ≤ 0.05, ***p* ≤ 0.01, and ****p* ≤ 0.001.


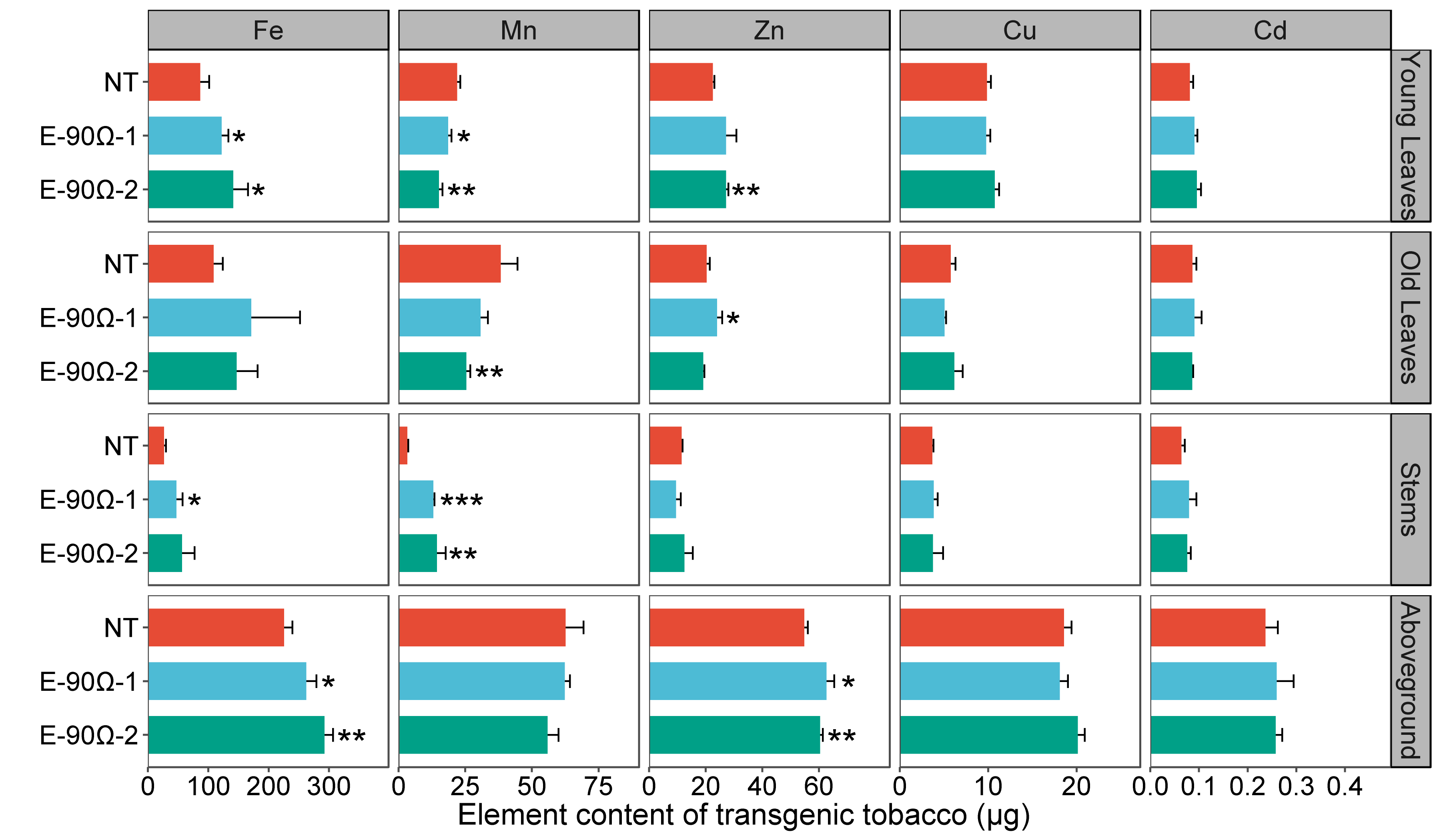


**Supplementary Figure 5.** Elements contents of *AhNRAMP1* transgenic tobacco grown on calcareous soil during the vegetative peroid. NT, non-transformed plants; E-90Ω, overexpressing *AhNRAMP1* transgenic plants induced by the E-90Ω promoter. The results are presented as the means ± SD of triplicate replicates. Asterisks indicate statistically significant differences with NT plants according to Student’s *t*-tests: **p* ≤ 0.05, ***p* ≤ 0.01, and ****p* ≤ 0.001.


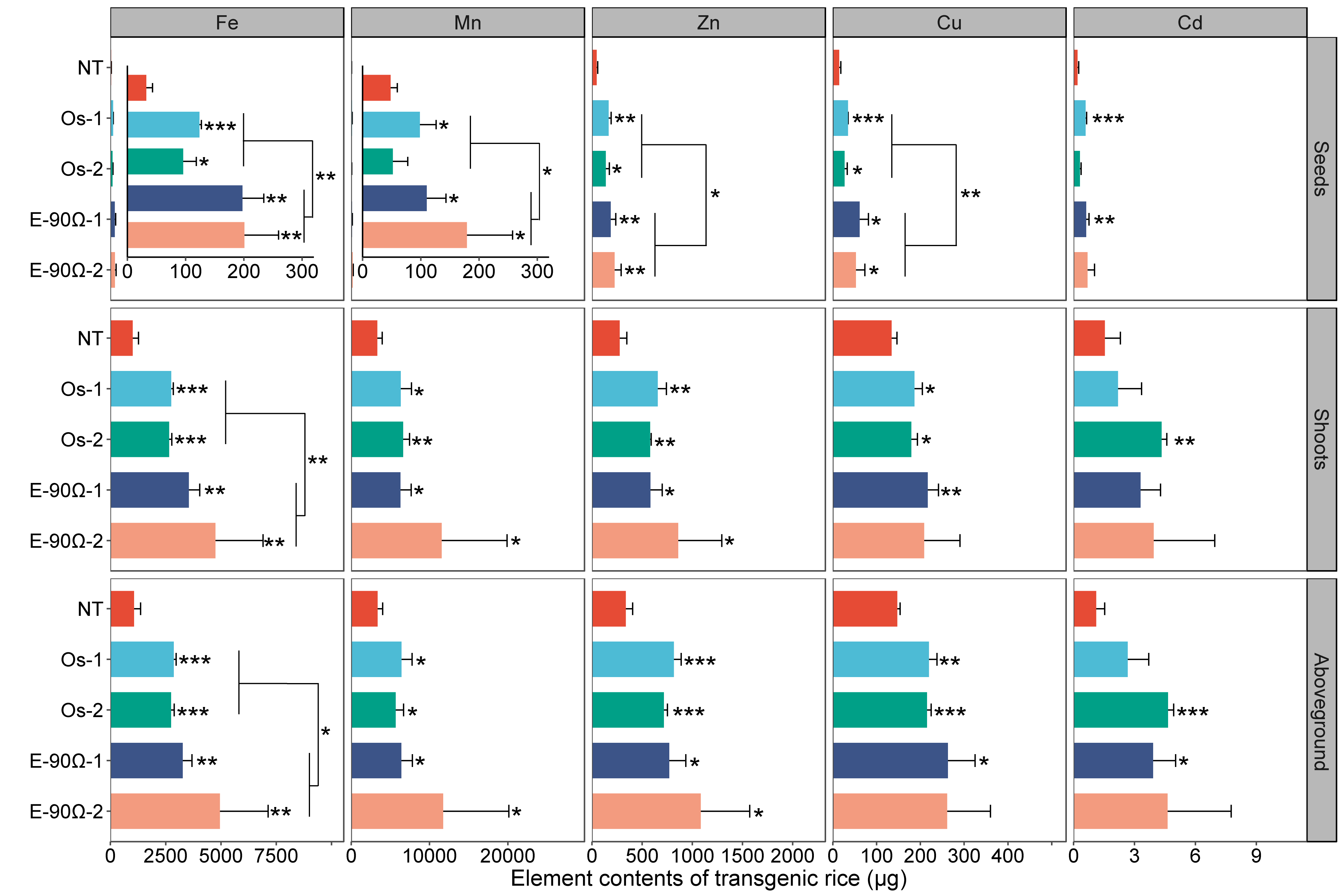


**Supplementary Figure 6.** Elements contents of mature *AhNRAMP1* transgenic rice grown on calcareous soil. NT, non-transformed plants; E-90Ω, overexpressing *AhNRAMP1* transgenic plants induced by the E-90Ω promoter; Os, *AhNRAMP1* transgenic rice induced by the *OsIRT1* promoter. The results are presented as the means ± SD of triplicate replicates. Asterisks indicate statistically significant differences with NT plants or between E-90Ω lines and *OsIRT1* promoter lines according to Student’s *t*-tests: **p* ≤ 0.05, ***p* ≤ 0.01, and ****p* ≤ 0.001.

## Supplementary Table

**Supplementary table S1. The primers for Quantitative real-time PCR**

| Gene name | Accession Number | Primer sequences |
| --- | --- | --- |
| *AhNRAMP1* | JQ581595 | Q*AhNRAMMP1*-F: 5'-CCTCATCACTGCCTTCGT-3'  Q*AhNRAMMP1*-R: 5'-ATTGCTGTGTTATCCTTGGTC-3' |
| *AhUbiquitin* | DQ887087.1 | Q*AhUbiquitin*-F: 5'-AAGCCGAAGAAGATCAAGCAC-3'  Q*AhUbiquitin*-R: 5'-GGTTAGCCATGAAGGTTCCAG-3' |
| *OsActin1* | KX302608.1 | Q*OsActin*-F: 5'-TGGACAGGTTATCACCATTGGT-3'  Q*OsActin*-R: 5'-CCGCAGCTTCCATTCCTATG-3' |
| *NtActin* | XM_009774717.1 | Q*NtActin*-F: 5'-AGTCCTCCTTACTGAAGCGCCT-3'  Q*NtActin*-R: 5'-GAATGGCAACGTACATAGCTGG-3' |
